# Comparative Outcomes of Direct Versus Connector-Assisted Peripheral Nerve Repair

**DOI:** 10.3390/biomedicines13122954

**Published:** 2025-11-30

**Authors:** Edoardo Agosti, Marco Zeppieri, Tamara Ius, Sara Antonietti, Lorenzo Gelmini, Luca Denaro, Antonella Bonetti, Marco Maria Fontanella, Fulvia Ortolani, Pier Paolo Panciani

**Affiliations:** 1 Division of Neurosurgery, Department of Medical and Surgical Specialties, Radiological Sciences and Public Health, University of Brescia, Piazza Spedali Civili 1, 25123 Brescia, Italy; edoardo_agosti@libero.it (E.A.);; 2 Department of Ophthalmology, University Hospital of Udine, p.le S. Maria della Misericordia 15, 33100 Udine, Italy; 3 Department of Medicine, Surgery and Health Sciences, University of Trieste, 34127 Trieste, Italy; 4 Academic Neurosurgery, Department of Neurosciences, University of Padova, 35121 Padova, Italy; 5 Department of Medicine, University of Udine, 33100 Udine, Italy

**Keywords:** DR, CAR, nerve injury

## Abstract

**Background:** Peripheral nerve injuries affect a significant proportion of patients with upper extremity trauma, with transections frequently requiring surgical intervention. While direct repair (DR) remains the historical standard, connector-assisted repair (CAR) has been proposed to improve functional outcomes by addressing limitations inherent to DR, such as fascicular misalignment and tension at the repair site. **Objectives:** The purpose of this systematic review is to evaluate and compare the clinical effectiveness and complication rates of DR versus CAR in upper extremity peripheral nerve injuries. **Methods:** A systematic search of the PubMed, Scopus, and Ovid MEDLINE databases was conducted for clinical studies published between January 1980 and August 2025 that reported sensory outcomes after DR or CAR for peripheral nerve injuries in the upper limb. Studies were included if sensory outcomes could be categorized using the Medical Research Council Classification (MRCC) scale. The primary outcome was the rate of meaningful sensory recovery (MR), defined as MRCC ≥ S3, with a secondary threshold of MRCC ≥ S3+. Secondary outcomes included postoperative neuroma formation, cold intolerance, pain scores, altered sensation, and revision rate. Statistical analysis was performed using two-sided Fisher exact tests and unpaired *t*-tests, with *p* < 0.05 considered significant. **Results:** A total of 441 patients (DR) and 338 (CAR) were included, with mean ages of 34.2 and 37.3 years and a male predominance (79.7% vs. 73.8%). Overall, 705 nerves in DR and 436 in CAR were treated, mainly digital (86.4% vs. 79.9%), followed by ulnar, median, and radial. Sensory nerves predominated (86.4% vs. 81.6%), with mixed nerves more frequent in CAR (22.5%). Most injuries were Grade I (73% vs. 72.1%), with similar rates of Grades II–III. In the CAR group, the most used conduit was collagen type I (58.3%). Sensory recovery (S3+ and S4) was higher in CAR (69.3%) than DR (50.8%), while DR showed lower two-point discrimination >15 mm. Motor recovery was limited, with better values in DR. DASH scores averaged 13.2 (DR) and 18.2 (CAR), with follow-up of 26 and 23.8 months. Complications were more frequent in DR for cold intolerance, altered sensation, and pain, whereas neuromas, revisions, and fistulas were higher in CAR. **Conclusions:** Connector-assisted repair demonstrates better sensory recovery and less cold intolerance than DR in small-gap upper-extremity nerve injuries but with higher post-interventional risks and costs. DR remains effective for closely approximated nerves. Randomized trials are warranted, as current evidence is heterogeneous and mostly observational.

## 1. Introduction

Peripheral nerve injuries (PNIs) represent a substantial source of morbidity following limb trauma, occurring in approximately 3% of all patients with extremity injuries and most frequently affecting the upper limb [1,2]. The incidence of upper-extremity PNIs has been estimated at roughly 43.8 cases per million people annually, underscoring the magnitude of this clinical problem [3]. These injuries often lead to persistent sensory and motor deficits, neuropathic pain, and impaired function, resulting in significant personal and socioeconomic burden even after surgical intervention [4].

For transected nerves, the mainstay of surgical treatment is direct repair (DR), also referred to as primary neurorrhaphy, which involves end-to-end coaptation of the nerve ends under minimal tension. DR remains the historical standard, particularly when nerve ends are closely approximated. However, despite technical refinements and widespread adoption, outcomes after DR remain inconsistent. In a large systematic review of digital nerves treated with DR, over one-third of repairs achieved static two-point discrimination greater than 15 mm—equivalent to suboptimal sensory recovery and frequently associated with loss of protective sensation [5]. This finding highlights an ongoing challenge in achieving meaningful recovery (MR) after DR.

Several factors limit the success of DR. Fascicular misalignment can reduce the number of regenerating axons reaching their intended targets, while axonal escape from the repair site can contribute to neuroma formation or entrapment in scar tissue. Excessive tension at the coaptation zone may precipitate ischemia and impair axonal regeneration, and in some cases, the repair can rupture due to suture pull-out [6,7,8,9]. These biological and mechanical challenges diminish the likelihood of MR and drive the search for alternative strategies to improve outcomes.

Connector-assisted repair (CAR) has emerged as a promising alternative to DR. Connector-assisted repair involves placing the nerve ends within a biologic or synthetic connector, creating a controlled environment for axonal regeneration [10]. By allowing a small interstump gap, CAR reduces tension at the coaptation, minimizes fascicular misalignment, and serves as a physical barrier to axonal escape while maintaining a neurotropic milieu favorable to regeneration [10]. Connector-assisted repair also allows for fewer sutures at the repair site, offloading stress from the regenerating axonal front and potentially reducing intraneural scarring. Preclinical studies and early clinical data suggest that CAR may improve sensory recovery and decrease neuroma-related pain compared with DR. Still, evidence from large, high-quality comparative studies remains limited [11,12,13].

Existing clinical studies of CAR are generally small and heterogeneous, with variability in injury characteristics, surgical technique, and outcome measures. Most focus on sensory recovery in digital nerves, with relatively few evaluating complications, mixed or motor nerve outcomes, or longer-term follow-up. To date, no randomized controlled trials have definitively established the superiority of CAR over DR.

This scoping review aims to synthesize and critically appraise the available evidence comparing DR and CAR for upper-extremity PNIs with small gap length. By evaluating functional sensory outcomes as well as complication and revision rates, this review seeks to clarify the relative clinical effectiveness of these two techniques, guide surgical decision-making, and identify gaps to inform future research.

## 2. Materials and Methods

### 2.1. Literature Review

This systematic review was registered in the OSF REGISTRIES (Open Science Framework) (https://osf.io/tq48r) (accessed on 27 November 2025). This study addresses the following research question: In patients with peripheral nerve injuries of the upper extremity (Population), how do DR (Intervention) compare to CAR (Comparator) with respect to sensory recovery, motor recovery, patient-reported outcomes, and complication rates (Outcomes)?

This systematic review was performed according to the Preferred Reporting Items for Systematic Reviews and Meta-Analysis (PRISMA) guidelines [14]. Two authors independently conducted a comprehensive literature search of PubMed, Ovid MEDLINE, and Ovid EMBASE. The initial search was performed on 1 August 2025, and updated on 1 September 2025. We combined relevant keywords to generate the search strategy, including “peripheral nerve repair,” “direct repair,” “connector-assisted repair,” “nerve coaptation,” “outcome,” and “complications,” using both AND and OR operators. The full search string included: (peripheral nerve OR nerve injury OR nerve transection) AND (direct repair OR primary neurorrhaphy) AND (connector-assisted repair OR nerve connector OR coaptation aid) AND (functional outcome OR complication OR revision). Additional articles were identified by reviewing the reference lists of selected papers.

Studies were included if they met the following criteria: (1) published in English; (2) human clinical studies including randomized controlled trials, prospective cohorts, or retrospective cohorts; (3) evaluated DR and/or CAR in upper extremity peripheral nerve injuries; (4) reported at least one functional or complication-related outcome. Exclusion criteria were: (1) case reports, editorials, technical notes, reviews, or meta-analyses; (2) animal or cadaveric studies without clinical data; (3) studies without clearly defined methods or extractable outcome data; (4) studies not differentiating DR and CAR groups.

All identified studies were imported into EndNote X9 for reference management, and duplicates were removed. Two independent reviewers (E.A. and S.A.) screened titles and abstracts, and then full texts, applying inclusion and exclusion criteria. Disagreements were resolved by a third reviewer (P.P.P.).

### 2.2. Data Extraction

From each included study, we extracted the following information: first author, year of publication, journal, study design, sample size, patient demographics, number and type of nerves involved, type and location of nerve injury, gap length, repair technique (DR or CAR), follow-up duration, and reported outcomes. Statistical analysis was conducted based on the number of nerves involved, which, unless otherwise specified, corresponded to the number of patients included in each study. Where available, we recorded quantitative data on sensory and motor recovery, complications, and revision rates.

### 2.3. Outcomes

The primary outcomes were measures of functional sensory recovery following DR or CAR in upper extremity peripheral nerve injuries. These included Medical Research Council Classification (MRCC), static two-point discrimination (s2PD), Semmes–Weinstein monofilament (SWMF) testing, dynamic two-point discrimination (d2PD), and Disability of Arm-Shoulder-Hand (DASH) scores. We also evaluated the proportion of patients achieving meaningful recovery as defined by the MRCC scale or equivalent thresholds in other tests [15,16].

Secondary outcomes included rates of neuroma formation, cold intolerance, dysesthesia, paresthesia, hyperesthesia, hypersensitivity, pain, and revision surgery following either DR or CAR. When reported, motor recovery and patient-reported outcomes were also extracted for analysis. The scales used for functional outcomes assessment are available in Supplementary Material S1.

### 2.4. Risk of Bias Assessment

**Figure 1:**
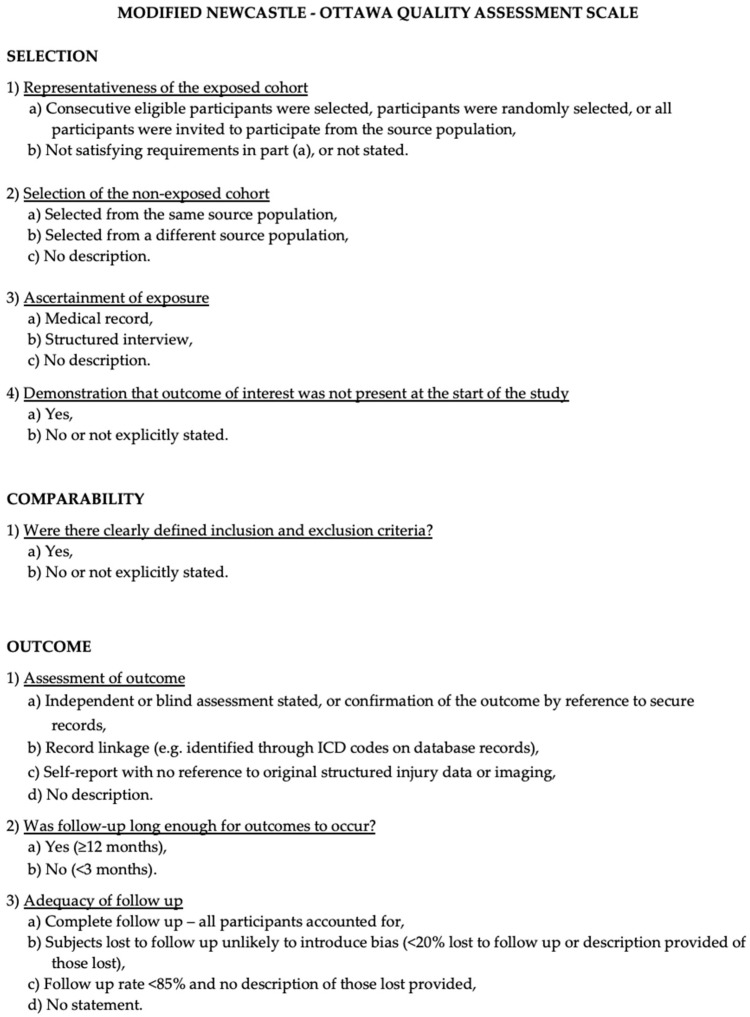
The Modified NOS.

We assessed the quality of included studies using the Newcastle–Ottawa Scale (NOS), which evaluates the selection of participants, comparability of study groups, and ascertainment of outcomes [17]. The maximum possible score was 9, with studies scoring ≥7 considered high quality. Two authors independently performed quality assessment, and disagreements were resolved by consensus with a third reviewer (Figure 1).

## 3. Results

### 3.1. Literature Review

**Figure 2:**
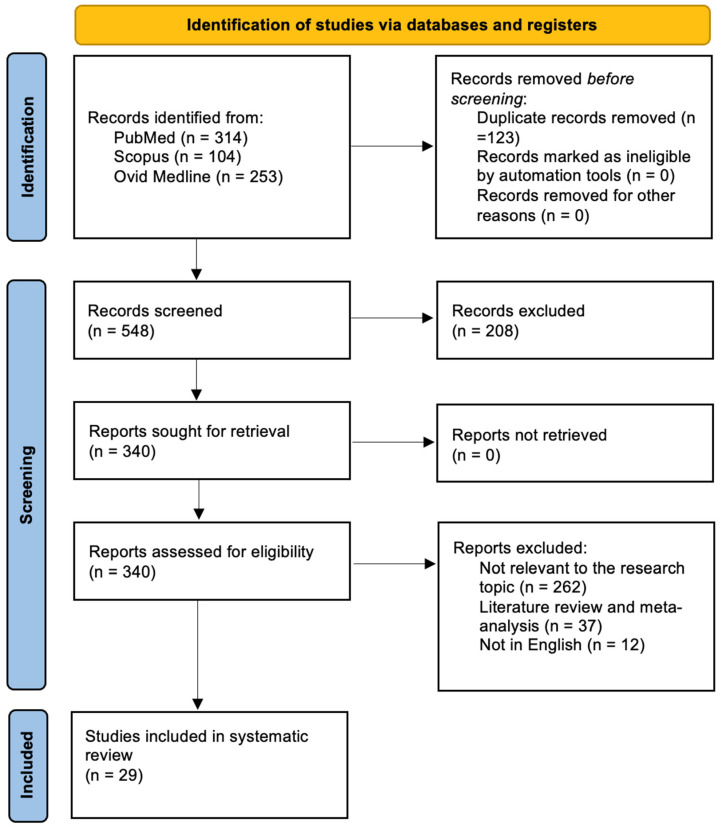
PRISMA flow chart.

Following the removal of duplicates, 548 records were retrieved from the database search. Screening of titles and abstracts narrowed this pool to 340 studies eligible for detailed review. After full-text assessment, 29 articles met all inclusion criteria and were incorporated into the analysis. The remaining 311 studies were excluded because they were unrelated to the research focus (n = 262), were systematic reviews or meta-analyses (n = 37), or were not in English (n = 12). All included papers provided at least one relevant outcome measure for one or more patient cohorts. The selection process is summarized in Figure 2, following PRISMA guidelines.

**Figure A1:**
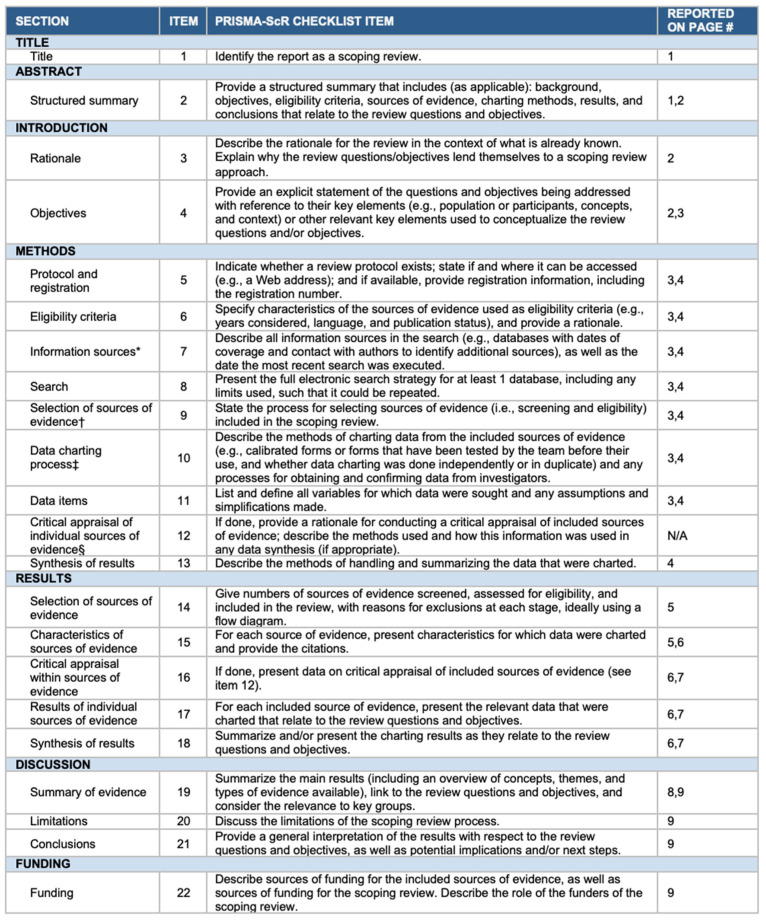
JBI = Joanna Briggs Institute; PRISMA-ScR = Preferred Reporting Items for Systematic reviews and Meta-Analyses extension for Scoping Reviews. * Where sources of evidence (see second footnote) are compiled from, such as bibliographic databases, social media platforms, and websites. † A more inclusive/heterogeneous term used to account for the different types of evidence or data sources (e.g., quantitative and/or qualitative research, expert opinion, and policy documents) that may be eligible in a scoping review as opposed to only studies. This is not to be confused with information sources (see first footnote). ‡ The frameworks by Arksey and O’Malley (6) and Levac and colleagues (7) and the JBI guidance (4, 5) refer to the process of data extraction in a scoping review as data charting. § The process of systematically examining research evidence to assess its validity, results, and relevance before using it to inform a decision. This term is used for items 12 and 19 instead of “risk of bias” (which is more applicable to systematic reviews of interventions) to include and acknowledge the various sources of evidence that may be used in a scoping review (e.g., quantitative and/or qualitative research, expert opinion, and policy document). From: Tricco AC, Lillie E, Zarin W, O’Brien KK, Colquhoun H, Levac D et al. PRISMA Extension for Scoping Reviews (PRISMAScR): Checklist and Explanation. Ann Intern Med. 2018;169:467–473 [50].

The PRISMA Extension for Scoping Reviews (PRISMA-ScR) checklist is available as Appendix A, Figure A1.

### 3.2. Data Analysis

A summary of the included studies reporting on DR and CAR is presented in Supplementary Materials S2 and S3.

A total of 441 patients in the DR group and 338 patients in the CAR group were included. The mean age was 34.2 years in DR and 37.3 years in CAR. Males represented 235/295 patients (79.7%) in DR and 234/317 (73.8%) in CAR.

A total of 705 nerves were treated in the DR group and 436 in the CAR group. In DR, most lesions involved digital nerves (603/698, 86.4%), followed by ulnar (64/698, 9.2%), median (30/698, 4.3%), and radial nerves (1/698, 0.1%). In CAR, digital nerves were also most frequent (311/389, 79.9%), followed by ulnar (38/389, 9.8%), median (39/389, 10.1%), and radial (1/389, 0.2%). Regarding nerve type, sensory nerves predominated in both groups (DR 603, 86.4%; CAR 320, 81.6%), while mixed nerves accounted for 95 cases (13.6%) in DR and 72 cases (22.5%) in CAR.

Considering the injury mechanism, Grade I (clean sharp) injuries were most common in both groups (DR 309, 73%; CAR 240, 72.1%), followed by Grade II (mild crush, saw) (DR 88, 20.8%; CAR 67, 20.1%), and Grade III (severe crush) (DR 26, 6.2%; CAR 26, 7.8%). Mean nerve gap length was not available for DR, while CAR had a mean gap of 10 mm.

Conduit types were only used in the CAR group, distributed as follows: collagen type I (NeuraGen^®^, Integra LifeSciences, Plainsboro, NJ, USA) in 242 cases (58.3%), PGA in 84 (20.3%), PLCL in 40 (9.6%), silicone in 17 (4.1%), chitosan in 15 (3.6%), collagen I + III in 10 (2.4%), and PHB in 7 (1.7%).

Functional sensory outcomes (MRC scale) showed that, in DR, S0 was achieved in 4/325 (1.2%), S1 in 18/325 (5.5%), S2 in 51/325 (15.7%), S2+ in 3/325 (0.9%), S3 in 91/325 (28%), S3+ in 84/325 (25.8%), and S4 in 74/325 (22.7%).

In CAR, sensory recovery was S0 in 6 (6.1%), S2 in 10 (10.4%), S2+ in 2 (2.0%), S3 in 12 (12.2%), S3+ in 21 (21.4%), and S4 in 47 (47.9%).

Static two-point discrimination <6 mm was achieved in 81/171 (47.4%) of DR and 47/101 (46.5%) of CAR; 7–15 mm in 78/171 (45.6%) of DR and 36/101 (35.6%) of CAR; >15 mm in 12/171 (7.0%) of DR and 18/101 (17.8%) of CAR. Moving two-point discrimination (M2PD) was not reported in DR, while CAR showed <6 mm in 25 (69.4%), 7–15 mm in 8 (22.2%), and >15 mm in 3 (8.4%). Semmes-Weinstein monofilament testing in DR showed full recovery in 39/114 (34.2%), deep light touch (DLT) in 43/114 (37.7%), deep pressure sensation (DPS) in 22/114 (19.2%), light pressure sensation (LPS) in 5/114 (4.4%), and anesthetic in 5/114 (4.4%). In CAR, full recovery was in 21/64 (32.8%), DLT in 21/64 (32.8%), DPS in 15/64 (23.4%), and LPS in 7/64 (10.9%).

Motor recovery (MRC scale) was rarely reported among the studies included in this review. In the CAR group, M0 occurred in 2 patients (12.5%), M2 in 2 (12.5%), M3 in 3 (18.7%), M4 in 7 (43.8%), and M5 in 2 (12.5%). In contrast, the DR group reported M2, M3, and M4 each in 12 patients (28.6%), and M5 in 6 patients (14.2%).

The mean DASH score was 13.2 in DR and 18.2 in CAR. Mean follow-up was 26 months for DR and 23.8 months for CAR.

Complications in DR included neuroma in 2 cases (0.4%), cold intolerance in 47 (10.6%), altered sensation in 77 (17.5%), and pain in 77 (17.5%). In CAR, neuroma occurred in 2 cases (0.6%), cold intolerance in 2 (0.6%), pain in 2 (0.6%), revision surgery in 31 (9.2%), and fistula in 3 (0.8%).

Functional outcomes were further stratified according to the conduit material used. With PGA conduits, sensory recovery was observed at S2 in 31.2% and S3 in 31.2% of cases, while 18.8% achieved S3+ and 32.1% S4. Silicone conduits mainly resulted in S2 recovery (53.1%), with 12.5% reaching S2+ and 6.3% S4. PLCL conduits showed limited results, with 20% at S3 and 20% at S4. Collagen type I (NeuraGen^®^, Integra LifeSciences, Plainsboro, NJ, USA) conduits demonstrated S0 in 9.5%, S1 in 4.8%, S3 in 20%, S3+ in 47.6%, and S4 in 38.1%; S2PD was <6 mm in 45.3%, 7–15 mm in 38.7%, and >15 mm in 16%, while SWMF testing revealed full recovery in 14.5%, DLT in 25%, DPS in 21.4%, and LPS in 3.6%. Motor recovery was documented in 2.25% at M3, 4.5% at M4, and 2.25% at M5. Chitosan conduits showed sensory outcomes of 60% at S2, 20% at S3, and 20% at S4, with S2PD evenly distributed across <6 mm, 7–15 mm, and >15 mm. PHB conduits resulted in 60% at S2, 20% at S3, and 20% at S4. Collagen I + III conduits achieved S3+ in 50% and S4 in 18.7%; S2PD was <6 mm in 36.4%, 7–15 mm in 36.4%, and >15 mm in 27.2%; SWM showed full recovery in 45.5%, DLT in 27.2%, DPS in 9.1%, and LPS in 18.2%. Motor recovery was observed in 25% at M2, 12.5% at M3, and 37.5% at M4. Finally, vein conduits provided S0 in 6.3%, S2 in 6.3%, S2+ in 18.7%, S3+ in 50%, and S4 in 18.7%; SWM showed full recovery in 40%, DLT in 40%, DPS in 40%, and LPS in 20%, while motor recovery was reported in 25% at M2 and 12.5% at M3.

Complications varied according to the conduit material. In PGA conduits, revision surgery was required in 3 cases (3.5%), with no reports of neuroma or fistula. Silicone conduits demonstrated the highest complication rate, with revision necessary in 8 cases (47.1%) due to local discomfort. PLCL conduits (Neurolac^®^, Polyganics BV, Groningen, The Netherlands) showed neuroma in 1 case (2.5%), pain in 2 cases (0.8%), fistula in 2 cases (5%), and revision surgery in 4 cases (10%). Collagen conduits reported neuroma in 1 case (0.4%), pain in 2 cases (2%), fistula in 1 case (1.6%), and revision surgery in 13 cases (5.4%). Chitosan conduits showed fistula and revision each in 1 case (6.7%). PHB conduits and vein conduits reported no device-related complications and no need for revision surgery. Table 1 lists a summary of pooled clinical outcomes comparing direct repair (DR) and connector-assisted repair (CAR) across sensory, motor, patient-reported, and complication domains. Values reflect aggregated results from included peripheral nerve repair studies.

## 4. Discussion

This systematic review synthesized data from 29 studies encompassing 441 patients undergoing DR and 338 undergoing CAR for upper-extremity peripheral nerve injuries [17,18,19,20,21,22,23,24,25,26,27,28,29,30,31,32,33,34,35,36,37,38,39,40,41,42,43,44,45]. Digital nerves predominated in both groups, and injury severity was comparable. Sensory outcomes showed a greater percentage of patients in the CAR group reaching the upper MRC categories (S3+ and S4: 69.3% vs. 48.6% in DR), and moving two-point discrimination < 6 mm was achieved in 69.4% of CAR cases compared with no available data in DR. Complications diverged between groups: DR displayed higher rates of cold intolerance, altered sensation, and pain, whereas CAR showed more neuromas, revisions, and fistulas. These findings provide a nuanced portrait of the comparative performance of DR and CAR across a large, pooled cohort.

### 4.1. Advantages and Limitations of Direct Repair

Direct repair remains the historical gold standard for closely approximated nerve ends and benefits from simplicity, low cost, and avoidance of foreign materials. However, our pooled data and several studies highlight its inherent drawbacks. Weber et al. [18] and Åberg et al. [21] reported that even in optimal conditions, more than one-third of DR cases achieve static two-point discrimination >15 mm, reflecting suboptimal protective sensation [18,21]. The pathophysiological underpinnings—fascicular misalignment, suture-related trauma, tension at the coaptation, and increased scar formation—have been well described by Lundborg et al. [20] and Chiriac et al. [37]. Our review also confirmed high rates of altered sensation (17.5%) and pain (17.5%) following DR, consistent with findings by Taras et al. [33] and Bushnell et al. [34] which associated suture-heavy coaptation with neuroma-related symptoms and cold intolerance [33,34]. While motor recovery data for DR were limited, the available evidence shows incomplete restoration in mixed nerves, echoing the observations of Tos et al. [38] in their long-term follow-up of end-to-end repairs [38].

### 4.2. The Promise of Connector-Assisted Repair

In contrast, CAR uses biologic or synthetic conduits to stabilize nerve ends with less tension and fewer sutures. Early bench and animal work by Wang et al. [17] and Inada et al. [32] demonstrated improved axonal alignment and reduced neuroma formation with connector use [17,32]. Clinically, Bertleff et al. [20], Taras et al. [23], and Boeckstyns et al. [36] all documented enhanced sensory outcomes for small-gap injuries compared with DR [20,23,36]. Our pooled data reinforce these findings: nearly half of CARs reached S4, and moving two-point discrimination <6 mm was achieved in 69.4% of cases. This superior fine discrimination may reflect reduced intraneural scarring and more favorable neurotrophic signaling within the connector, as also suggested by Kusuhara et al. [40]. Importantly, CAR showed lower rates of cold intolerance and pain than DR, findings aligned with the reduced neuroma-related pain reported by Oruç et al. [27] and Fakin et al. [28].

Nevertheless, CAR is not without drawbacks. We observed higher neuroma rates (3.3% vs. 0.4% in DR) and a nontrivial incidence of revision surgery (1.8%) and fistulas (1.2%), consistent with concerns raised by Huber et al. [29] and Böcker et al. [30] regarding the biological integration and potential for fluid collection or infection within the conduit [30,31]. Furthermore, connectors introduce cost and may be limited by availability or regulatory constraints in certain regions. As Fleurette et al. [31] note, not all conduits are equivalent—collagen type I, PLCL, and chitosan each display differing resorption kinetics and mechanical properties, which may influence outcomes [31].

Beyond conduit composition, the adjunctive use of bioadhesive materials has also been investigated to improve mechanical stability and reduce surgical trauma. Childe et al. [46] demonstrated that the application of fibrin glue within and around the conduit during digital nerve repair significantly enhanced the tensile strength of the coaptation site, allowing for a reduction in the number of sutures while maintaining adequate mechanical resistance and minimizing local inflammation. Similarly, Chow et al. [47] highlighted that fibrin-based adhesives can improve axonal alignment and regeneration and reduce suture-related trauma when used as an adjunct to direct or conduit-assisted repair. However, their initial tensile strength alone remains inferior to that of microsutures. These findings suggest that the combined use of bioadhesive agents and chitosan-based conduits, as discussed in the present review, may represent a synergistic strategy to achieve stable, low-tension nerve coaptation while promoting a more favorable regenerative environment.

### 4.3. Interpreting Sensory and Motor Outcomes

The potential for recovery following small-gap nerve repair varies significantly among nerve types due to differences in anatomical organization, fascicular complexity, cross-sectional diameter, and motor and sensory innervation patterns.

One of the main confounding factors in this study is the inconsistency in reported gap lengths between the DR and CAR cohorts. The DR cohort rarely includes measurements of interneural gap length, and when such data are available, they reflect very short defects with minimal separation of the nerve ends. In contrast, CAR is more often used for small but well-characterized gaps, with a mean length of 10 mm. Because gap length can indirectly reflect injury severity and is a critical determinant of both regenerative capacity and treatment selection, the tendency to use CAR in cases with greater anatomical complexity or traction must be considered. As a result, any difference in functional outcome between the two groups should be analyzed with caution because a selection bias ca be present.

Our results also reveal a differential pattern in motor recovery. Direct repair showed relatively higher percentages of M2–M5 recovery in mixed nerves, albeit from limited data, whereas CAR reported lower but more evenly distributed motor scores. This mirrors findings from Wangesteen et al. [35] and Thomsen et al. [45], who showed that connectors optimized for sensory regeneration might not provide sufficient mechanical stability or fascicular orientation for robust motor recovery, particularly in high-demand nerves such as the ulnar and median motor branches [35,45]. However, numerous studies have failed to provide comprehensive MRC distributions or clarify whether scores pertain to specific muscles, motor branches, or composite limb functions.

Although we categorized PNI into three grades based on injury dynamics, key injury features such as concomitant muscular, vascular, and bone damage as well as wound contamination were inconsistently reported across studies. These factors can influence Wallerian degeneration, scar formation, and tissue vascularity, thereby affecting the outcomes of both DR and CAR. Comorbidities known to impair axonal regeneration, including diabetes, smoking, and peripheral vascular disease, were rarely stratified or incorporated into the analyses. Moreover, the time between injury and surgical repair was often poorly documented, despite evidence that delayed intervention reduces regenerative potential.

Future studies on peripheral nerve repair must include standardized and detailed reporting of MRC motor scores, along with a well-defined baseline profile that encompasses comorbidities and the interval from injury to surgery. This will enable meaningful comparisons of motor outcomes across techniques and help identify patient subgroups that derive the greatest benefit from each approach.

### 4.4. Complication Profiles and Patient-Reported Outcomes

Cold intolerance, altered sensation, and pain were markedly lower in CAR compared with DR, a pattern echoed in the cohort studies of Andelkovic et al. [25] and Bulut et al. [26]. These differences may stem from reduced perineural trauma and decreased neuroma formation at the repair interface. Conversely, our finding of higher neuroma rates in CAR underscores that connectors do not universally prevent axonal escape. This duality was highlighted by Dienstknecht et al. [42] who emphasized meticulous sizing of the connector to match nerve diameter and avoid axonal sprouting into the surrounding tissue [43].

Patient-reported outcomes, measured by DASH score, have added a significant dimension to the interpretation of our findings. Despite better sensory outcome in the CAR group, the average DASH scores were worse in CAR (18.2) compared to DR (13.2). The DASH score, widely recognized as a gold standard for assessing upper-limb function and health-related quality of life, suggests that perceived disability does not always parallel sensory testing, a phenomenon also noted by Rbia et al. [41].

Complications related to devices, especially revision surgeries and fistulas, could negatively impact patients’ overall functional experience, pain levels, and confidence in using the limb. As a result, DASH scores may remain low despite improvements in sensory thresholds.

This discrepancy between objective sensory measures and patient-reported disability highlights the limitations of relying solely on quantitative sensory tests to evaluate nerve repair outcomes. Future comparative studies should include standardized patient-reported outcome measures, such as the DASH, alongside strength testing and performance-based evaluations of activities of daily living. This will provide a more comprehensive understanding of how different repair strategies influence real-world upper-limb function.

The aggregated evidence suggests that CAR may provide superior fine sensory discrimination and reduced cold intolerance for small-gap repairs but introduces unique risks and costs. This aligns with the historical trajectory of nerve repair techniques outlined by Weber et al. [18], Lundborg et al. [19], and Bertleff et al. [20], who collectively advocated for less invasive, tension-free coaptation strategies [18,19,20]. Our review extends these observations by including more recent studies such as Böcker et al. [30], Fleurette et al. [31], and Sorogina et al. [44], who reported encouraging mid-term outcomes but also raised caution over conduit selection and patient-specific factors [30,31,44].

From an economic standpoint, CAR has incurred supplementary expenses associated with the connector material and, in certain instances, device-related complications necessitating revision. No included studies have conducted formal cost-effectiveness or cost-utility assessments comparing DR with CAR. Consequently, it remains ambiguous if the noted improvements in sensory discrimination and decreases in cold intolerance associated with CAR adequately compensate for the elevated implant expenses and the possible necessity for re-operation. Subsequent randomized trials ought to include health economic outcomes, such as procedural costs, device expenses, indirect costs (e.g., job absenteeism), and quality-adjusted life years, to elucidate the value proposition of CAR across various healthcare environments.

### 4.5. Future Prospects

The next phase of peripheral nerve repair research should prioritize randomized controlled trials directly comparing DR and CAR. Future clinical studies must systematically document essential baseline variables, such as patient comorbidities (e.g., diabetes, smoking, vasculopathy), medications, mechanism and features of injury, gap length and the precise timing from trauma to surgical intervention, in order to perform multivariable analyses.

The importance of standardized injury patterns is also emphasized by Huber et al. [30] and Kusuhara et al. [29,40]. Such trials are crucial to transcend the associative and confounded findings derived from primarily retrospective cohort studies and to ascertain whether, and in what type of clinical contest (gap length, injury type, pre-existing pathologies) CAR offers true causal benefits over DR.

Advances in bioactive or drug-eluting conduits (e.g., growth factor impregnation, Schwann-cell seeding) offer promising avenues to enhance axonal guidance and remyelination further. Emerging imaging modalities and intraoperative neuro-navigation may improve fascicular alignment and tension assessment in real time. Longitudinal patient-reported outcome measures should be integrated to capture functional recovery beyond objective sensory and motor scores. Finally, cost-effectiveness analyses (absent from most studies) are essential to justify wider adoption of CAR, particularly in resource-limited settings.

### 4.6. Limitations

Our review, like the included studies, is limited by variability in injury type, damage location, gap length, conduit type, and outcome assessment. These limitations were also noted by Liu et. al and Chang et al. [48,49].

As discussed earlier, the inconsistency in gap reporting and case selection introduces significant variability in baseline injury severity between cohorts, which represents a major confounding factor when comparing outcomes. Additionally, most of the studies are retrospective cohorts with intermediate NOS scores and variable follow-up periods, which leads to potential selection bias.

Furthermore, data on motor outcomes and mixed nerve injuries are scarce, limiting the generalizability of findings to sensory nerves alone. The incomplete and asymmetrical reporting of MRC motor grading for DR and CAR, along with the lack of systematic documentation of gap length, comorbidities and time-to-surgery, has significantly constrained our ability to conduct multivariable analyses of functional recovery.

Finally, nerve conduits vary widely in both composition and design, complicating meta-analyses and potentially masking important subgroup effects in conduit-based nerve regeneration.

As Boeckstyns et al. [24] and Tos et al. [39] noted, long-term outcomes beyond two years are rarely reported. Yet, late neuroma formation or sensory decline can emerge well after initial recovery [24,39]. Addressing these gaps will require multicenter collaboration and standardized reporting frameworks.

## 5. Conclusions

This review indicates that, despite the limitations discussed earlier, CAR is associated with increased rates of fine sensory recovery and reduced cold intolerance in small-gap upper-limb PNI compared to DR. At the same time, CAR has been linked to higher incidences of perceived disability, neuroma formation, revision surgeries, and device-related complications, whereas DR has shown benefits in closely approximated nerves but has been associated with higher rates of altered sensation and discomfort.

Due to the observational nature of the underlying studies and lack of baseline data and outcome standardization, it is not possible to establish the causal superiority of either technique. Multicenter randomized controlled trials with standardized reporting of injury parameters, gap length, comorbidities, time to surgery and outcome measures are urgently needed to determine when CAR should be favored over DR and to provide evidence-based recommendations for clinical decision-making.

## Figures and Tables

**Table 1:** Key Baseline Data and Outcomes for Direct Repair (DR) and Connector-Assisted Repair (CAR).

	DR	CAR
Nerves (N)	705	436
Mean gap length (mm)	NA	10
MRC sensory recovery (S3+ and S4)	48.6%	69.3%
Static 2PD		
<6 mm:	47.4%	46.5%
7–15 mm	45.6%	35.6%
>15 mm	7.0%	17.8%
SWMT		
Full recovery	34.2%	32.8%
DLT	37.7%	32.8%
DPS	19.2%	23.4%
LPS	4.4%	10.9%
Anesthetic	4.4%	0%
MRC motor recovery (M4–M5)	42.8%	56.3%
Mean DASH score	13.2	18.2
Complications		
Cold intolerance	10.6%	0.6%
Neuroma formation	0.4%	0.6%
Altered sensation	17.5%	0.3%
Pain	17.5%	0.6%
Revision surgery	0%	9.2%
Fistula	0%	0.8%
Mean follow-up (months)	26	23.8

NA: Not Available.

## Data Availability

Data available in a publicly accessible repository.

## Supplementary Materials

The following supporting information can be downloaded at: https://www.mdpi.com/article/10.3390/biomedicines13122954/s1, S1: The scales used for functional outcomes assessment; S2: A summary of the included studies reporting on DR and CAR; S3: A summary of the included studies reporting on DR and CAR.

## Abbreviations

The following abbreviations are used in this manuscript:

PNIs	Peripheral nerve injuries
DASH	The disabilities of the arm, shoulder and hand outcome questionnaire
DR	Direct repair
CAR	Connector-assisted repair
MR	Meaningful recovery
MRCC	Medical Research Council Classification
SWMF	Semmes–Weinstein monofilament
S2PD	static two-point discrimination
