# Supplementary material for: Comparative Outcomes of Direct Versus Connector-Assisted Peripheral Nerve Repair
Source: Biomedicines. 2025 Nov 30;13(12):2954. doi: 10.3390/biomedicines13122954 (PMC12730310; doi:10.3390/biomedicines13122954)
Supplement: Supplementary file 1 [file biomedicines-13-02954-s001.zip › S1.pdf]

Table: Highet scale modified by Dellon et al. for classification of sensory nerve recovery

S0	Absence of sensibility in the autonomous zone of the nerve
S1	Recovery of deep cutaneous pain sensibility within the autonomous zone of the nerve
S2	Recovery of superficial pain and tactile sensibility within the autonomous area of the nerve, persisting paraesthesia
S3	Return of superficial cutaneous pain and tactile sensibility throughout the autonomous area with disappearance of any previous over response; static two point-discrimination: >15 mm
S3+	Return of sensibility as in S3; in addition there is some recovery of two-point discrimination within the autonomous area; static two-point discrimination: 7–15 mm
S4	Complete recovery, static two point discrimination <7 mm

Table: Highet scale modified by Dellon et al. for classification of motor nerve recovery

M0	No contraction
M1	Return of perceptible contraction in the proximal muscle
M2	Return of perceptible contraction in the proximal and distal muscles, active movement without gravity
M3	Active movement against gravity, return of function in both proximal and distal muscles of such a degree that all important muscles are sufficiently powerful to act against gravity
M4	Active movement against gravity, return of function in both proximal and distal muscles of such a degree that all important muscles are sufficiently powerful to act against gravity
M5	Full recovery in all muscles

Table: Dellon classification for 2PDT

Measurement	Interpretation
<6 mm	Normal
6 mm to 15 mm	Alterated
>15 mm	Poor
No point perceived	Anesthesia

Table: Mackinnon–Dellon clinical scheme for Semmes Weinstein monofilament test

2.83	Normal
3.61	Diminished light touch (DLT)
4.31	Diminished protective sensation (DPS)
4.56	Loss of protective sensation (LOSS PS)
6.65	Deep pressure only
No response	Anesthetic
