# Supplementary material for: Comparative Outcomes of Direct Versus Connector-Assisted Peripheral Nerve Repair
Source: Biomedicines. 2025 Nov 30;13(12):2954. doi: 10.3390/biomedicines13122954 (PMC12730310; doi:10.3390/biomedicines13122954)
Supplement: Supplementary file 1 [file biomedicines-13-02954-s001.zip › S2.pdf]

Author and year	Patients (N)	Anamnestic data		N° nerve	Nerve (N, %)	Nerve type	Injury type	Mean Gap Length (Range, mm)	Functional Outcome						FU (range)
		Male (N, %)	Mean Age (range)						Sensory				Motor	DASH score	
									MRCC	S2PD	M2SD	SWM	MRCC		
Wang et al., 1996	N/A	N/A	N/A	74	Digital	sensory	Clean sharp (29, 43.9%); mild crush (37, 56.1%)	N/A	N/A	≤ 7 mm: 36, 49%	≤ 7 mm: 59, 80%	N/A	-	N/A	>= 12 months
Weber et al., 2000	N/A	N/A	N/A	74	Digital (74, 100%)	Sensory	N/A	4.3 mm	S4 (32, 42.8%)	N/A	N/A	N/A	-	N/A	8.1 ± 5.0 mo (3–12)
Ludborg et al., 2004	13	13, 100%	33 (15–64)	13	Median (6, 46.2%) Ulnar (7, 53.8%)	Mixed (sensory+ motor)	clean sharp (13, 100%)	N/A	S2 (3, 25%) S2+ (3, 25%) S3 (3, 25%) S3+ (3, 25%)	N/A	N/A	N/A	Motor domain score: 0.87 (0.76–0.94) Manual muscle test: 0.80 (0.53–1) Grip strength: 0.95 (0.89–1)	N/A	12 months (3-60)
Bertleff et al., 2005	13	10, 75%	38	13	Digital (13, 100%)	sensory	clean sharp (13, 100%)	N/A	N/A	N/A	N/A	N/A	-	N/A	12 months
Åberg M et al., 2009	6	6, 100%	26 (15-41)	7	Ulnar (6, 85.7%) median (1, 14.3%)	Mixed	Clean sharp (7, 100%)	N/A	S2 (5, 83.3%) S0 (1, 16.7%)	N/A	N/A	N/A	N/A	N/A	18 months

Artiaco et al., 2010	7	4, 57.14%	45 (20-62)	7	Digital (7, 100%)	Sensory	N/A	N/A	S3+ (7,100%)	12.7 mm (8–18)	N/A	DLT (2, 29%) DPS (5, 71%)	-	N/A	35 (8-60)
Boeckstyns et al., 2013	13	2, 15.4%	33 (21-65)	21	Median (13, 61.9%) Ulnar (8, 38.1%)	Mixed	Clean sharp (21, 100%)	N/A	N/A	N/A	N/A	N/A	Rosen score: 0.60	N/A	24 months
Basar et al., 2014	42	34, 80.95%	30.68	42	Ulnar (42, 100%)	Mixed	Clean sharp (28, 66.7%) Severe crush injury (14, 33.3%)	N/A	S2 (6,14.29%) S3 (12, 28.57%) S3+ (13, 30.95%) S4 (11, 26.19%)	N/A	N/A	N/A	M2 (12, 28.57%) M3 (12, 28.57%) M4 (12, 28.57%) M5 (6, 14.29%)	25.07 (mean value)	31.55
Andelkovic et al., 2016	150	126, 84%	34.3	193	Digital (193, 100%)	Sensory	N/A	N/A	S0 (3, 1.6%) S1 (18, 9.3%) S2 (37, 19.2%) S3 (76, 39.4%) S3+ (41, 21.3%) S4 (18, 9.3%	N/A	N/A	N/A	-	Mean 9.0	30 months (12-60)
Bulut et al., 2016	63	N/A	36.4 (11-62)	96	Digital (96, 100%)	Sensory	Clean sharp (52, 54.2%) Mild crush (44, 45.8%)	N/A	N/A	< 6mm (26, 27%) 7-15 mm (61, 64%) > 15 mm (9, 9%)	N/A	Full: (31, 32%), DLT (38, 40%), DPS (17, 18%), LPS (5, 5%), Anesthetic 5 (5%)	-	N/A	21.4 months

Fakin et al., 2016	83	N/A	N/A	93	Digital (93, 100%)	Sensory	Clean sharp (77, 82.8%) Mild crush (5, 5.4%) Severe crush (11, 11.8%)	N/A	N/A	Mean 10.6 mm	N/A	Mean 2.7	-	N/A	42 months (12-72)
Oruc et al., 2016	19	15, 78.9%	38.4 (15-62)	38*	Digital (38, 100%):	Sensory	Clean sharp (38, 100%)	N/A	≤S3 (5, 13%) S3+ (20, 53%) S4 (13, 34%)	<6mm (10,36%), 7-15 mm (15,54%), > 15 mm (3,11%)	N/A	N/A	-	N/A	16.4 months (12-19)
Huber et al., 2017	14	11, 78.5%	38.6	15	Digital (15, 100%)	Sensory	Clean sharp (15, 100%)	N/A	N/A	N/A	N/A	N/A	-	Mean 5.6	6-36 months
Böcker et al., 2022	7	6, 86%	45.43 ± 5.85	7	N/A	N/A	Mild crush (2, 29%) Clean sharp (5,71%)	12.80	N/A	7.9 ± 1.1 m	5.3 ± 0.6 mm	3.7 ± 0.5	N/A	N/A	12
Fleurette et al, 2022	11	8, 72.7%	9.7 (5-15)	12	Median (10, 83.3%) Radial (1, 8.3%) Ulnar (1, 8.3%)	Mixed	Clean sharp (11, 91.7%) Severe crush (1, 8.3%)	N/A	N/A	< 6 mm (9, 81.8%) 7–15 mm (2, 18.2%)	N/A	Full (8, 72.7%) DLT (3, 27.3%)	M3-M5	Mean QuickDASH: 5.99/100 (range 0–18.18)	92.4 months (24-168)

Table 1: direct repair: N/A= not a, Full = full sensation, LPS: light protective sensation; DLT: diminish light touch; DPS: diminished protective sensation

* **8 digits / 38 nerves** (Group-1 unilateral: 18 digits/18 nerves; Group-2 bilateral: 10 digits/**20** nerves)

Author and year	Patients (N)	Anamnestic data		N° nerve	Nerve	Nerve type	Injury type	Connector material	Mean Gap Length (Range, mm)	Functional Outcome						FU (range)
		Male (N, %)	Mean Age (range)							Sensory				Motor	DASH score Mean (range)	
										MRCC	S2PD	M2PD	SWM	MRCC		
Weber et al., 2000	N/A	N/A	N/A	62	Digital (62, 100%)	Sensory	N/A	PGA	7.0 ± 5.6 (0–30, ≤3 cm)	S4 (27, 43.5%)	N/A	N/A	N/A	-	N/A	9.4 ± 4.4 mo (3–12)
Ludborg et al., 2004	17	13, 76.5 %	32 (12–72)	17	Median (7, 41.2%) Ulnar (10, 58.8%)	Mixed	Clean sharp (17, 100%)	silicone tube	3-5 mm	S2 (5, 31.3%) S2+ (2, 12.5%) S3 (5, 31.3%) pazienti S3+ (3, 18.8%) S4 (1, 6.3%)	N/A	N/A	N/A	Motor domain = 0.73 (0.71–0.91) MMT (thumb/finger muscles) = 0.53 (0.53–0.90) Grip strength = 0.90 (0.85–0.98)	N/A	12 months (3-60)
Inada et al., 2004	2	1, 50%	59 (56-62)	2	Digital (1, 50%) Superficial peroneal nerve (1, 50%)	Sensory	N/A	PGA and collagen coated	20-65 mm	N/A	3–5 mm (1, 50%) > 10 mm (1, 50%)	N/A	N/A	-	N/A	5-6 months
Bertleff et al., 2005	17	13, 76%	43	21	Digital (21, 100%)	Sensory	Clean sharp (21, 100%)	PLCL	8 mm 4-20 mm	N/A	N/A	N/A	N/A	-	N/A	12 months
Taras et al., 2008	2	N/A	N/A	2	Digital (2, 100%)	Sensory	Clean sharp (2, 100%)	NeuraGen®	15-20 mm	N/A	6mm (1, 50%),	N/A	N/A	-	N/A	6-8 months

								type I collagen			N/A (1, 50%)					
Bushnell et al., 2008	12	8, 88.9 %	33 (18-50)	12	Digital (12, 100%)	sensory	Clean sharp (6, 50%) Mild crush (4, 33.3%) Severe crush (2, 16.7%)	Neuragen Type I collagen	10-20 mm	S4 (4, 44%) S3+ (5, 56%)	3mm (1, 8.3%) 5mm (4, 33.3%) 7mm (2, 16.7%) 8mm (1, 8.3%) 9mm (2, 16.7%) 13 (1, 8.3%)	N/A	F (5, 55.6%) DLT (2, 22.2%) DPS (1, 11.1%) LPS (1, 11.1%)	-	N/A	15 (12-22) months
Lohmeyer et al., 2009	15	10, 71.4 %	38 (12-66)	16	Digital (16, 100%)	Sensory	Clean sharp (13, 86.7%) Mild crush (2, 13.3%)	Neuragen Type I collagen	12,7 mm (6-18)	S0 (2, 16.7%) S2 (1, 6.7%) S3+ (5, 33.3%) S4 (4, 26.7%)	4mm (2, 13.3%) 5mm (1, 6.7%) 6mm (1, 6.7%) 7 mm (2, 13.3%) 9 mm (1, 6.7%) 10 mm (1, 6.7%) > = 15 mm (4, 26.7%)	N/A	N/A	-	N/A	12 months
Åberg M et al., 2009	6	5, 83%	36 (15-58)	7	Ulnar (5, 83%) Median (2, 17%)	Mixed	Clean sharp (7, 100%)	PHB	N/A	S2 (3, 60%) S3 (1, 20%) S4 (1, 20%)	N/A	N/A	N/A	N/A	N/A	18 months
Wangesteen et al., 2010	96	67, 70%	33 (7-79)	126	Digital (82,65.1%); non-digital small-caliber (23, 18.3%).; Large-	Sensory and mixed	Clean sharp (72, 57%) Mild crush (37, 29%)	Neuragen Type I collagen	12.8 mm (2.5–20); digital subset 11.7	N/A	N/A	N/A	N/A	N/A	N/A	8 months

					caliber (21, 16.7%)		Severe crush (15, 12%)		mm (2.5–20)							
Thomsen et al., 2010	10	3, 30%	30, (16-49)	10	Digital (10, 100%)	Sensory	Clean sharp (10, 100%)	Revolnerv1 TypeI+II collagen	11.25 mm (5–20)	N/A	4mm (4, 36.4%)<br>8mm (1, 9.1%)<br>11 mm (1, 9.1%)<br>12 mm (2, 18.2%)<br>15 mm (2, 18.2%)<br>16 mm (1, 9.1%)	N/A	Full (5, 45.5%)<br>DLT (3, 27.3%)<br>DPS (1, 9.1%)<br>LPS (2, 18.2%)	-	19.3 (0–39)	11.8 months
Taras et al., 2011	19	N/A	N/A	22	Digital (22, 100%)	sensory	Clean sharp (22, 100%)	Neuragen Type I collagen	12mm (5-17)	N/A	3mm (1, 4.5%)<br>4mm (6, 27.3%)<br>5 mm (4, 18.2%)<br>6 mm (2, 9.1%)<br>7 mm (3, 13.6%)<br>> = 8 mm (7, 31.8%)	3 mm (5, 22.7%)<br>4 mm (5, 22.7%)<br>5 mm (6, 27.3%)<br>6 mm (2, 9.1%)<br>7 mm (3, 13.6%)<br>8 mm (2, 9.1%)	N/A	-	N/A	20 months
Chiriac et al., 2012	23	20, 87%	39.5 (17-61)	25	Digital (12, 42.9%)<br>median (5, 17.9%)<br>ulnar (5, 17.9%)<br>inter-digital (1, 3.6%)	Mixed Sensory	N/A	PLCL Neurolac™	10.3	N/A	24.96 mm	N/A	Mean: LPS	Grip strength: mean 64.6%	N/A	21.9 (3-45)

					musculocutaneous (1, 3.6%)<br>radial (1, 3.6%)											
Tos et al., 2012	16	12, 75%	37 (17-60)	16	Digital (8, 50%)<br>median (4, 25%),<br>Ulnar (4, 25%)	Sensory and Mixed	N/A	vein conduit filled with fresh skeletal muscle	sensory mean 12 mm; mixed mean 25 mm	S0 (1, 6.3%)<br>S2 (1, 6.3%)<br>S3 (3, 18.7%)<br>S3+ (8, 50%)<br>S4 (3, 18.7%)	N/A	N/A	DLT (6, 40%)<br>DPS (6, 40%)<br>LPS (3, 20%)	M0 (2, 25%)<br>M2 (2, 25%)<br>M3 (1, 12.5%)<br>M4 (3, 37.5%)	N/A	53 months
Boeckstyns et al., 2013	18	11, 61.1 %	37, (21-66)	23	Median (11, 47.8%)<br>Ulnar (12, 52.2%)	Mixed	Clean sharp (23, 100%)	Neuragen type I Collagen	< 6 mm	N/A	N/A	N/A	N/A	Rosen score: 0.75	N/A	24 months
Means et al., 2016	9	6, 66.7 %	38 ± 12 (20-53)	13	Digital (13, 100%)	Sensory	N/A	N/A	13.2 mm (5-20 mm)	S0 (3, 23.1%)<br>S3+ (3, 23.1%)<br>S4 (7, 53.8%)	4 mm (3, 23.1%)<br>5 mm (2, 15.4%)<br>6 mm (3, 23.1%)<br>10 mm (2, 15.4%)<br>16 mm (3, 23%)	4 mm (3, 23.1%)<br>5 mm (2, 15.4%)<br>6 mm (2, 15.4%)<br>7 mm (2, 15.4%)<br>8 mm (1, 7.7%)<br>16 mm (3, 23.1%)	Full (2, 15.4%)<br>DPS (2, 15.4%)<br>LPS (1, 7.7%)<br>Deep pressure only (3, 23.1%)<br>DLT (5, 38.5%)	N/A	8	9 months
Huber et al., 2017	10	10, 100%	45.6	11	Digital (11, 100%)	Sensory	N/A	Neuragen type I Collagene	15 mm	N/A	N/A	N/A	N/A	-	mean 25.0	6-36 months
Kusuhara et al., 2019	20	15, 75%	47 (18-79)	20	Digital (20, 100%)	Sensory	Clean sharp (12, 60%), mild crush	PGA	16.7 mm (1-50 mm)	N/A	Mean 8.6 ± 1.2 mm	N/A	N/A	-	N/A	13 months

							(5, 25%), severe crush (3, 15%)									(12-15)
Rbia et al., 2019	19	19, 100%	38	19	Digital (19, 100%)	Sensory	Clean sharp (12, 63%)<br>Severe crush (7, 37%)	Neuragen type I Collagen	14 mm	N/A	< 6mm (9, 48%)<br>7-15 mm (5, 26%)<br>>15 mm (5, 26%)	N/A	Full (9, 48%)<br>DLT (5, 26%)<br>DPT (5, 26%)	-	N/A	12 months
Böcker et al., 2022	15	10, 67%	35.9± 3.3	15	N/A	N/A	Clean sharp (11, 73%)<br>Mild crush (3, 20%)<br>Severe crush (1, 7%)	Chitosan	13.25 ± 1.90	N/A	10.9 ± 1.3 mm	6.6 ± 1.4 mm	Mean: DLT	N/A	N/A	12 months
Dienstknecht et al., 2023	9	8, 88.9%	25 (10-41)	9	Median (9, 100%)	Mixed	Clean sharp (9, 100%)	Neuragen Type I collagen	10-20 mm	N/A	< 6 mm (2, 22.2%)<br>6–10 mm (4, 44.4%)<br>10 mm (3, 33.4%)	N/A	N/A	M3 (2, 25%)<br>M4 (4, 50%)<br>M5 (2, 25%)	6 (0-25)	21 months
Sorogina et al., 2025	3	3, 100%	50	3	Ulnar (2, 66.4%)<br>Median (1, 33.3%)	Mixed	Clean sharp (3, 100%)	Neuragen Type I collagen	47 mm (30-60 mm)	N/A	N/A	N/A	N/A	M>4 (3, 100%)	ulnar: 4.2 → 1.7 (12 months)<br>median: 9.2 → 0 (12 months).<br>ulnar: 28.3 → 23.3 (12 months)	6-12 months

Table 2: connector-assisted repair PHB= Polyhydroxybutyrate, PGA= polyglycolic acid, PLCL = poly-DL-lactide-ε-caprolactone; Full = full sensation, LPS: light protective sensation; DLT: diminish light touch; DPS: diminished protective sensation
