# Supplementary material for: Comparative Outcomes of Direct Versus Connector-Assisted Peripheral Nerve Repair
Source: Biomedicines. 2025 Nov 30;13(12):2954. doi: 10.3390/biomedicines13122954 (PMC12730310; doi:10.3390/biomedicines13122954)
Supplement: Supplementary file 1 [file biomedicines-13-02954-s001.zip › S3.pdf]

TOTAL PATIENTS N				DR	CAR
				**441**	**338**
**ANAMNESTIC DATA**	Age (mean, range)			34.2	37.3
	Male (N, %)			235/295, 79.7%	234/317, 73.8%
**N° NERVES**				705	436
**NERVES**	Digital			603/698, 86.4%	311/389, 79.9%
	Median			30/698, 4.3%	39/389, 10.1%
	Ulnar			64/698, 9.2%	38/389, 9.8%
	Radial			1/698, 0.1%	1/389,0.2%
**NERVES TYPES**	Sensory			603, 86.4%	320, 81.6%
	Mixed			95, 13.6%	72, 22.5%
**INJURY TIPE**	Grade I (clean sharp)			309, 73%	240, 72.1%
	Grade II (mild crush, saw)			88, 20.8%	67, 20.1%
	Grade III (severe crush)			26, 6.2%	26, 7.8%
**MEAN GAP LENGHT**				N/A	10 mm
**CONDUIT TYPE**	PGA			-	84, 20.3%
	silicone			-	17, 4.1%
	PLCL			-	40, 9.6%
	Collagen type I (Neuragen)			-	242, 58.3%
	Chitosan			-	15, 3.6%
	PHB			-	7, 1.7%
	Collagen I+III			-	10, 2.4%
**FUNCTIONAL OUTCOME**	**Sensory (N, %)**	MRC	S0	4/325, 1.2%	6, 6.1%
			S1	18/325, 5.8%	
			S2	51/325, 16.4%	10, 10.4%
			S2+	3/325, 0.9%	2, 2.0%
			S3	91/325, 29.3%	12, 12.2%
			S3+	84/325, 27%	21, 21.4%
			S4	74/325, 23.8%	47, 47.9%
		S2PD	< 6 mm	81/171, 47.4%	47/101, 46.5%
			7-15 mm	78/171, 45.6%	36/101, 35.6%

			> 15 mm	12/171, 7.0%	18/101, 17.8%
		M2PD	< 6 mm	N/R	25, 69.4%
			7-15 mm	N/R	8, 22.2%
			> 15 mm	N/R	3, 8.4%
		SWM	Full	39/114, 34.2%	21/64, 32.8%
			DLT	43/114, 37.7%	21/64, 32.8%
			DPS	22/114, 19.2%	15/64, 23.4%
			LPS	5/114, 4.4%	7/64, 10.9%
			Anesthetic	5/114, 4.4%	N/R
	**Motor (N, %)**	MRC	M0	N/R	2, 2.8%
			M2	12, 28.6%	2, 2.8%
			M3	12, 28.6%	3, 4.2%
			M4	12, 28.6%	7, 9.7%
			M5	6, 14.2%	2, 2.8%
	**DASH SCORE (mean)**			13.2	18.2
**FOLLOW UP (months)**				26	23.8
**COMPLICATIONS**	**NEUROMA (N, %)**			2, 0.4%	2, 0.6%
	**COLD INTOLLERANCE (N, %)**			47, 10.6%	2, 0.6%
	**ALTERATED SENSATION**			77, 17.5%	N/A
	**PAIN**			77, 17.5%	2, 0.6%
	**REVISION**			N/R	31, 9.2%
	**FISTULA**			N/R	3, 0.8%
